# Nitrate Mercury in Uterine Hemorrhage

**Published:** 1874-02

**Authors:** 


Nitrate of Mercury in Periodical Uterine Hæmorrhages.—Dr. Joulin has for some time treated the periodical hæmorrhages, which occur between the catamenia, by means of the acid nitrate of mercury, which he applies to the cervical canal by means of a glass rod moistened with the medicinal fluid. The effect is incontestable, and the new method causes neither pain nor accidents. It is necessary, however, to make a vaginal injection at once, without which there would be severe and persistent smarting. If the cervical canal is contracted, a preparative dilatation is to be made. If the rod does not hold a sufficient quantity of the nitrate, Joulin uses a steel rod on which a little cotton is wound. The little tampon, after having been well moistened with the nitrate, is carried into the cervical canal and then quickly removed. Joulin asserts that he has hardly ever failed in his object by this method, and that he has thus cured hæmorrhages of long duration which had proved rebellious to other modes of treatment. Good results have also been attained in persistent leucorrhœas.—*Jour. de Joulin* and *Gaz. Med. Ital. Lombard, No.* 35, 1873.

